# British Axial Spondyloarthritis Inception Cohort (BAxSIC): a protocol for a multicentre real-world observational cohort study of early axial spondyloarthritis

**DOI:** 10.1093/rap/rkae087

**Published:** 2024-07-26

**Authors:** Jake Weddell, Stephanie R Harrison, Alexander N Bennett, Karl Gaffney, Gareth T Jones, Pedro M Machado, Jonathan Packham, Raj Sengupta, Sizheng Steven Zhao, Stefan Siebert, Helena Marzo-Ortega

**Affiliations:** NIHR Leeds Biomedical Research Centre, Leeds Teaching Hospitals NHS Trust, Leeds, UK; Leeds Institute of Rheumatic and Musculoskeletal Medicine, University of Leeds, Leeds, UK; NIHR Leeds Biomedical Research Centre, Leeds Teaching Hospitals NHS Trust, Leeds, UK; Leeds Institute of Rheumatic and Musculoskeletal Medicine, University of Leeds, Leeds, UK; Leeds Institute of Cardiovascular and Metabolic Medicine, University of Leeds, Leeds, UK; Leeds Institute of Data Analytics, University of Leeds, Leeds, UK; National Heart and Lung Institute, Faculty of Medicine, Imperial College, London, UK; DMRC, Academic Department of Military Rehabilitation, Loughborough, UK; Norfolk and Norwich University Hospital NHS Foundation Trust and Norwich Medical School, University of East Anglia, Norwich, UK; Aberdeen Centre for Arthritis and Musculoskeletal Health (Epidemiology Group), University of Aberdeen, Aberdeen, UK; Department of Neuromuscular Diseases and Centre for Rheumatology, University College London, London, UK; Unit of Population and Lifespan Sciences, University of Nottingham, Nottingham, UK; Royal National Hospital for Rheumatic Diseases, Royal United Hospitals, Bath, UK; Centre for Therapeutic Innovation, Department of Life Sciences, University of Bath, Bath, UK; Division of Musculoskeletal and Dermatological Sciences, University of Manchester, Manchester, UK; School of Infection and Immunity, College of Medical, Veterinary and Life Sciences, University of Glasgow, Glasgow, UK; NIHR Leeds Biomedical Research Centre, Leeds Teaching Hospitals NHS Trust, Leeds, UK; Leeds Institute of Rheumatic and Musculoskeletal Medicine, University of Leeds, Leeds, UK

**Keywords:** axial spondyloarthritis, rheumatology, diagnostic delay, inception cohort, virtual follow-up

## Abstract

**Objectives:**

Timely diagnosis remains a challenge in axial SpA (axSpA). In addition, data are scarce on the impact of diagnostic delay and disease progression in affected individuals. The British Axial Spondyloarthritis Inception Cohort (BAxSIC) study aims to investigate the impact of newly diagnosed axSpA, the natural history of the disease and the effect of diagnostic delay on disease outcomes.

**Methods:**

BAxSIC is a prospective, multicentre, observational study. Eligible participants are adults (≥16 years of age), with a physician-confirmed diagnosis of axSpA in the 6 months prior to study entry, recruited from secondary and tertiary rheumatology centres in the UK. Participants will be followed up for 3 years, with in-person visits at baseline and 24 months. In addition, patient self-reported assessments will be recorded remotely via the online electronic case report form (eCRF) at 6, 12, 18, 30 and 36 months.

**Results:**

The first patient was enrolled in BAxSIC in June 2023. Recruitment is currently ongoing and is planned to end in June 2026. Initial results will be available in 2027. Since opening, the trial has undergone two protocol amendments.

**Conclusion:**

The BAxSIC study is the first inception cohort designed to investigate the impact of diagnostic delay on clinical presentation and long-term functional outcomes in patients with axSpA in the UK. With an innovative, patient-led virtual longitudinal data collection model, data generated from this study will help inform and improve the care of people newly diagnosed with axSpA.

**Trial registration:**

ClinicalTrials.gov (http://clinicaltrials.gov), NCT05676775.

Key messages

- BAxSIC is a large UK-based inception cohort of newly diagnosed axSpA patients utilising virtual follow-up.
- BAxSIC aims to investigate the impact of diagnostic delay in axSpA and long-term functional outcomes.

## Introduction

Axial SpA (axSpA) is a chronic, immune-mediated condition characterized by inflammation in the axial skeleton and associated with extramusculoskeletal manifestations (EMMs). In the UK, an estimated 220 000 people live with axSpA, with a prevalence of 0.3–1.2% depending on the definition utilized [1]. The prototypic phenotype formerly known as AS is defined by the presence of new bone formation, leading to fusion in the spine and SI joints detected on radiographs [radiographic axSpA (r-axSpA)], which often manifests late in the disease course and not in all patients with axSpA. The advent of MRI at the turn of this century facilitated earlier diagnosis, prior to the development of new bone in the spine and SI joints and in those in whom new bone formation does not occur [non-radiographic (nr-axSpA)]. In parallel with these diagnostic advances, the development of biologic and targeted synthetic DMARDs (bDMARDs and tsDMARDs) have greatly improved quality of life and function in people with axSpA by improving symptoms of back pain, fatigue and stiffness [2]. However, their impact on disease progression and long-term outcomes is less clear [3].

Diagnostic delay is common in axSpA [4], and is worse in females than males [5]. In the UK, a recent report from the National Axial Spondyloarthritis Society (NASS) highlighted a mean time from symptom onset to diagnosis of 8.5 years [6]. Causes of diagnostic delay in axSpA are multifactorial and include the insidious onset of the disease, poor public and healthcare worker awareness, the absence of diagnostic serum biomarkers [7] and the reliance on clinician diagnosis based on the history, imaging and laboratory findings. The impact of this delay on patients is poorly researched, with a few small-scale studies primarily focusing on male patients with r-axSpA [8]. A recent study showed a higher burden of disease with the development of uveitis and inflammatory bowel disease associated with longer diagnostic delay [9]. Further research into the impact of diagnostic delay on the individual’s initial presentation and on the wider axSpA population is therefore vital to improving patient care and outcomes.

Hypothesizing that diagnostic delay leads to worse long-term functional outcomes and affects the overall disease course of axSpA, we aimed to assess the impact of diagnostic delay on clinical presentation and long-term outcomes, including work participation, quality of life and function in axSpA.

## Methods

### Study design

The BAxSIC study (https://baxsic.uk) is a prospective inception cohort study (Supplementary material, available at *Rheumatology Advances in Practice* online) recruiting patients with newly diagnosed axSpA from secondary and tertiary rheumatology centres across the UK. The study protocol was developed by members of the Executive Committee of the British Society for Spondyloarthritis (BRITSpA, https://www.britspa.co.uk/), who are co-investigators for the study and members of the Trial Management Group (TMG). There is no minimum sample size required for the study due to the exploratory nature of the primary outcome.

### Study population

**Figure 1.**
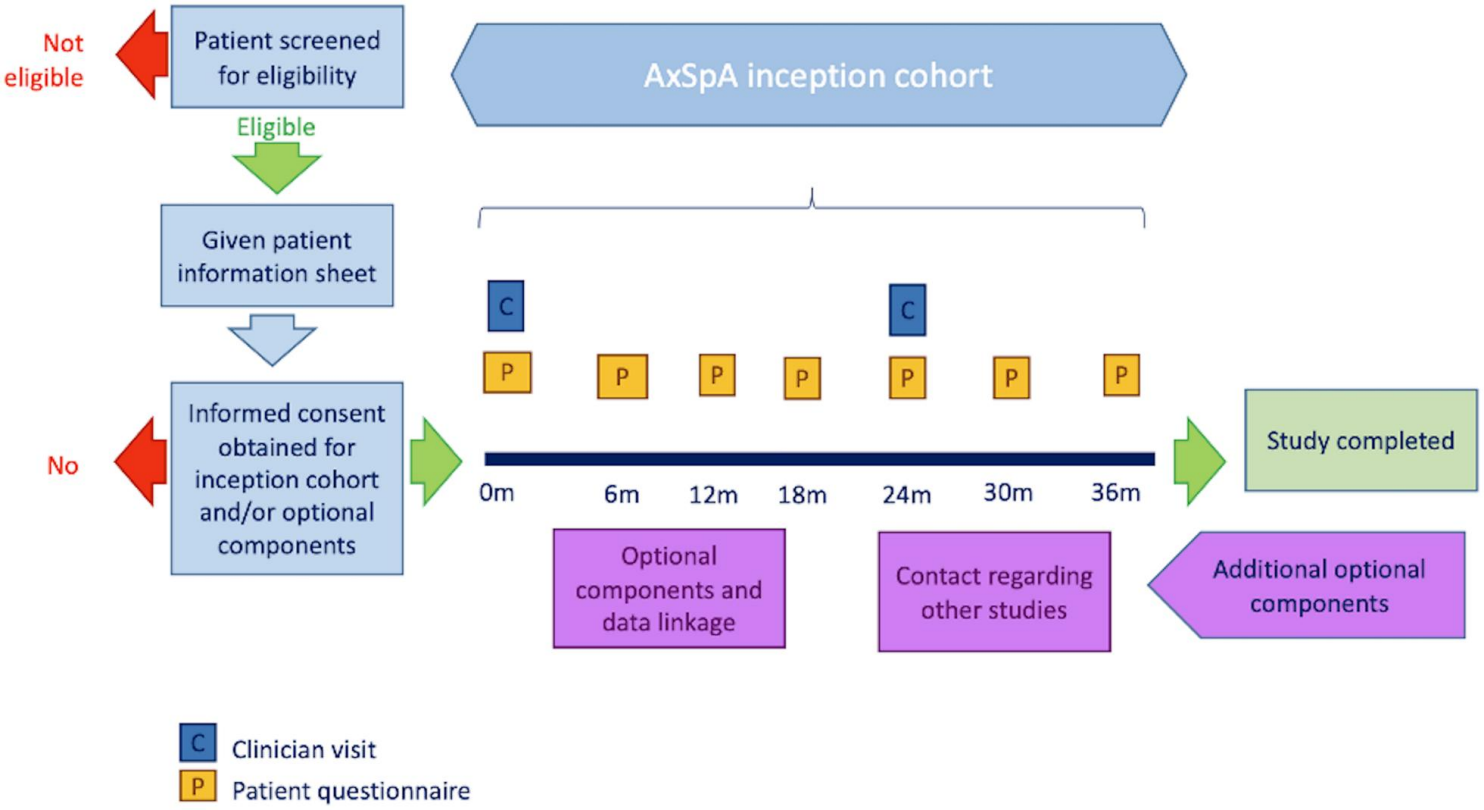
Schematic diagram of the BAxSIC study assessments

Patients are primarily recruited from rheumatology clinics. Eligible participants are consecutive patients who are ≥16 years of age with a diagnosis of axSpA made by a consultant rheumatologist within the 6 months prior to recruitment and able to give informed consent (Fig. 1). The exclusion criteria are age <16 years, unable to provide informed consent in English (despite efforts to facilitate informed consent through a Trust-appointed interpreter) or deemed in any other way to be unable to give informed consent. Recruitment to this study is based on a collaborative effort between multiple UK secondary and tertiary centres with the help of BRITSpA. Recruitment will last for 3 years with a total follow-up duration of 36 months (Fig. 1).

### Baseline procedures

Consenting participants will attend an in-person baseline visit where data on date of onset of symptoms, date of confirmed diagnosis, sociodemographic characteristics, general health, family history and data relating to symptom onset and diagnosis date will be collected (Table 1). In addition, the following information available from routine medical records will be recorded: medication data including utilization of NSAIDs, conventional synthetic DMARDs, bDMARDs, tsDMARDs, analgesics and steroids. Laboratory data from HLA-B27 testing (ever) and CRP or ESR values available on the electronic health records for up to 6 months from diagnosis and thought to be related to axSpA in the opinion of the investigator will be recorded. Available imaging data from routine anteroposterior (AP) radiographs of the pelvis/SI joints and MRI of the SI joints and spine performed as part of routine clinical practice up to 12 months prior to study entry will be collected together with data from the report by local readers (either radiologist or rheumatologist) regarding the presence or absence of radiographic sacroiliitis in accordance with modified New York (mNY) criteria [10] or a positive MRI according to Assessment of SpondyloArthritis international Society (ASAS) definitions [11].

**Table 1.** Summary schedule of study assessments

Month ±28 days	0	6	12	18	24	30	36
Visit number	1	2	3	4	5	6	7

Clinician-/allied health professional–initiated assessments							
Full informed consent	X						
Confirmation of ongoing consent				X			
Inclusion/exclusion criteria	X						
Study ID/site	X						
NHS number 1	X						
Demographic data							
Sex	X						
Age at study entry	X						
Marital status	X						
Ethnic group	X						
Highest level of education attained	X						
Employment status	X			X			
Post code (for deprivation score)	X						
Alcohol intake (units per week)	X			X			
Smoking status (current, ex, never)	X			X			
History/symptoms							
Onset of back pain symptoms (date/duration of)	X						
Onset of other SpA-related symptoms (date/duration of)	X						
History/diagnosis							
Date of first rheumatology appointment	X						
Date of diagnosis (by consultant rheumatologist)	X						
Meets mNY criteria	X			X			
Meets ASAS criteria	X			X			
Relevant past medical history: psoriasis, psoriatic nail changes, uveitis, IBD, enthesitis, dactylitis, arthritis, costochondritis/date of onset	X			X			
Family history: AS, axSpA, psoriasis, IBD, inflammatory arthritis	X			X			
Relevant orthopaedic surgery (arthroplasty or spinal)	X			X			
Current evidence of: psoriasis, psoriatic nail changes, uveitis, IBD, enthesitis, dactylitis, arthritis, costochondritis/date of onset	X			X			
Events of special interest	X			X			
Medication (previous and current)							
NSAIDs	X			x			
DMARDs	X			X			
Other (including steroids, analgesics)	X			X			
Laboratory and imaging							
HLA-B27	X						
CRP (mg/l)	X			X			
ESR (mm/h)	X			X			
Imaging (SI joints X-ray/MRI)	X			X			
ASDAS-CRP	X			X			
Participant-initiated assessments							
Height (cm)	X			X			
Weight (kg)	X			X			
Events of special interest		X	X		X	X	X
Patient-reported outcomes							
Total spinal pain score	X	X	X	X	X	X	X
Patient global assessment of disease activity	X	X	X	X	X	X	X
BASDAI	X	X	X	X	X	X	X
BASFI	X	X	X	X	X	X	X
4D-AS questionnaire	X						
ASAS-HI	X	X	X	X	X	X	X
EQ-5D-5L	X	X	X	X	X	X	X
Work productivity and activity impairment and occupation	X	X	X	X	X	X	X
Anxiety and depression, PROMIS short form 4a	X	X	X	X	X	X	X
Fatigue, PROMIS short form fatigue 8a	X	X	X	X	X	X	X
Fibromyalgia, Wolfe questionnaire	X	X	X	X	X	X	X

The investigator will assess the current Axial Spondyloarthritis Disease Activity Score (ASDAS), determine whether the participant fulfils the mNY [10] or ASAS classification criteria [11] for axSpA and examine for any current evidence of psoriasis, uveitis, IBD, enthesitis, dactylitis, peripheral arthritis or costochondritis. Participants will complete questionnaires to assess function, work participation, disease activity, fatigue, comorbidities, overall health and quality of life (Table 2).

**Table 2.** Patient-reported outcome measures and questionnaires

Total spinal pain [20]
Patient global assessment of disease activity [21]
BASDAI [22]
BASFI [23]
4D-AS [24]
ASAS Health Index [25]
EuroQol 5 dimension, 5-level [26]
Work Productivity and Activity Impairment Questionnaire: Specific Health Problem (version 2.0) [27]
PROMIS Depression Short Form 4a [28]
PROMIS Anxiety Short Form 4a [29]
PROMIS Fatigue Short Form 8a [30]
Fibromyalgia, Wolfe questionnaire [31]

### Follow-up procedures

At 6-month intervals (months 6, 12, 18, 30 and 36) participants will be asked to complete electronic data collection sheets and questionnaires (Table 2) via e-mail or text message. These will collect data regarding disease activity and EMMs in addition to patient-reported outcome measures. Participants will be sent up to three electronic reminders via the Research Electronic Data Capture (REDCap) system to complete each visit. Each visit will have a window of 28 days (±14 days). Participants may request paper versions, if required.

At 24 months (Fig. 1), participants will be invited for a further in-person review to collect data on medication use related to axSpA, employment status, alcohol and smoking status and comorbidities. Participants will be examined for any extra-axial features of SpA and data from routinely performed MRIs, X-rays and CRP/ESR will be collected. Patient reported outcomes and questionnaire data will also be collected at this visit (Table 2).

### Events of special interest

Only relevant predefined events of special interest (pregnancy and development of EMMs) will be recorded on the electronic case report form (eCRF).

#### Pregnancy

Participants who have reported a pregnancy will be asked to complete a questionnaire within 6 months after the due date. This will collect information on the date of conception, previous pregnancies, pre-conception counselling, assisted conception, complications in pregnancy, gestation length, pregnancy outcome, complications in labour and delivery, postpartum complications and infections, breastfeeding and perinatal complications.

#### EMMs

The development of EMMs will be recorded by either the physician/researcher (month 24) or the participant (months 6, 12, 18, 30 and 36). These include a new diagnosis of psoriasis, uveitis and IBD.

### Data storage and analysis

Data will be recorded in a standardized eCRF to ensure uniformity across all participating clinical sites using REDCap software, created by Vanderbilt University (Nashville, TN, USA). Data will be collected, stored and processed in compliance with the General Data Protection Regulation and the Data Protection Act 2018. Statistical analysis will be performed by researchers and statisticians in the Leeds Teaching Hospitals Trust and University of Leeds and other co-investigators in participating centres. Initially, descriptive statistics will be used to describe the demographics of patients newly diagnosed with axSpA. Regression analysis and other statistical methods will be applied to determine the impact of diagnostic delay on baseline characteristics as appropriate. As longer-term data become available, analysis will evaluate the impact of diagnostic delay on comorbidities, work participation and functional outcomes, describing the natural history and impact of newly diagnosed axSpA and identifying potential predictors of poor long-term outcome and b/tsDMARD therapy use.

### Ethics approval and consent to participate

Ethical approval for the BAxSIC study was obtained from the Wales Research Ethics Committee 7 (22/WA/0311) in 2022. The BAxSIC study is sponsored by the Leeds Teaching Hospitals Trust (RR21/146198) and is adopted on the National Institute Health and Research (NIHR) musculoskeletal study portfolio (54230) being eligible for the NIHR Associate Principal Investigator Scheme. All participants provide informed, written consent to take part in the study. Ethical approval for same-day and remote consent has been granted. The BAxSIC protocol has undergone two amendments, with the latest version (3.0) dated 20 December 2023. Protocol amendments will be disseminated to each study site for reconsent and updating of participants.

### Trial oversight

The BAxSIC study is coordinated by a TMG that consists of a chief investigator, deputy chief investigator, co-investigators and study coordinator. The TMG will act as the Data Access Committee. Independent oversight of the study will be conducted by the Trial Steering Committee, expected to meet yearly and which will include a lay individual, lay clinician independent of the study research team and at least one patient representative. In addition, an External Advisory Committee of three international experts has been appointed to advise the study investigators on maximizing the usefulness of the inception cohort for research purposes and to align opportunities for data collection with international studies or potential collaborations.

### Dissemination of results

Study results and outcomes will be disseminated to participants and healthcare professionals through a variety of channels, including journal publications, presentations at conferences and social media. The BAxSIC website (https://baxsic.uk/) will be updated with easily accessible study updates for participants on a regular basis and a newsletter will be circulated to participating sites on a quarterly basis.

## Discussion

Delay in diagnosis remains a significant unmet need in axSpA. A recent systematic review and meta-analysis confirmed a mean delay of 6.7 years from symptom onset to diagnosis, which did not improve when results were stratified by year of publication [4]. The BAxSIC study was designed to be the first inception cohort to explore the impact of diagnostic delay on clinical presentation, natural history and long-term outcomes in patients with newly diagnosed axSpA in the UK. Previous inception cohort studies such as GESPIC [12] and DESIR [13] have greatly improved the understanding of axSpA in recent years. However, significant gaps remain in our knowledge of areas identified by patients as priorities, including work participation, mental health and the effects of diagnostic delay [14]. The BAxSIC study aims to improve our understanding of these areas.

Diagnostic delay is common in axSpA, however, there is limited knowledge of how this affects patients and their disease course. A 2020 literature review identified 21 articles showing that a longer time to diagnosis was consistently associated with higher disease activity, poorer physical function, heightened anxiety and depression and greater healthcare costs [15]. However, 15 of these publications were confined to patients with r-axSpA with no inclusion of nr-axSpA, reporting on limited numbers, with just 4 of them having >200 participants and addressing a primarily male population, with 75% of participants being male [15]. A recent retrospective study [9] including >3000 patients from several Latin America and European centres reported an association between longer diagnostic delay and the *de novo* appearance of uveitis and IBD in r-axSpA/AS, highlighting the need to enhance diagnostic strategies to shorten the time from first symptom to diagnosis in SpA. Yet the small number of studies, overall low patient numbers and sex bias in the r-axSpA populations reported to date in the literature highlight the need for further studies to explore the impact of diagnostic delay upon the whole axSpA spectrum.

Identifying the impact of delay upon clinical presentation, natural history and long-term outcomes may allow for improved screening and diagnosis, assist in identifying patients at a higher risk of poor outcomes and comorbidities and improve our understanding of key areas of patient priority [14].

The development of b/tsDMARD therapies has greatly improved the treatment options available to patients, improving quality of life and short-term outcomes [16]. However, the effect of diagnostic delay on b/tsDMARD response is unclear [17]. Much of the data regarding b/tsDMARDs comes from clinical trials, which recruit a narrow subset of the axSpA population and are of insufficient length to address long-term outcomes such as EMMs and disease progression [18]. Therefore, longitudinal, real-world observational studies, such as BAxSIC, are required to explore these outcomes in patients with axSpA.

The BAxSIC study predominantly utilizes a virtual follow-up approach, with the majority of outcomes of interest being patient reported. The lack of specific procedures and minimal additional in-person study visits is aimed to minimize the burden on participants and thereby maximize the number of participating sites and patients recruited in a cost-effective manner. However, this minimally disruptive approach may lead some participants to disengage from the study and therefore fail to complete the virtual questionnaires. Therefore, encouraging ongoing participation is vital to the study’s success. A previous axSpA cohort study utilizing a virtual follow-up to capture outcomes in axSpA has been successfully performed in the UK with good rates of follow-up data completion [19]. Yet a number of factors were considered at the time of study design in order to minimize attrition over time. These include the short time to completion of virtual visits, which averages 10 min, and a window of ±14 days at each end of the follow-up visit’s target, providing a total of 28 days for visit completion. In addition, up to three electronic reminders are sent automatically by the REDCap system, including a standard e-mail containing the survey link and unique QR code. The study has recently been granted ethical approval for the use of telephone text messaging and remote consent, which is also expected to minimize disruption and encourage engagement of study participants. A dedicated section for participants within the study website includes an easy-to-understand timeline (https://baxsic.uk/patient-involvement-in-baxsic/).

## Conclusion

The BAxSIC study will provide real-world data in an inception cohort of axSpA, with participants remotely contributing validated patient-reported outcome measures routinely used in clinical practice. Although based in the UK, the results from this study will be of international relevance, evaluating the scope and impact of newly diagnosed axSpA, the impact of diagnostic delay upon initial presentation and long-term outcomes and the effect of bDMARDs on the disease course, all identified by researchers and patients as areas of unmet need in this disease.

## Supplementary Material

rkae087_Supplementary_Data

## Data Availability

Anonymized study data can be made available to other academic researchers upon reasonable request, subject to the approval of the BAxSIC Data Access Committee, which will consist of the TMG and include clinical and patient representation.
