# Supplementary material for: British Axial Spondyloarthritis Inception Cohort (BAxSIC): a protocol for a multicentre real-world observational cohort study of early axial spondyloarthritis
Source: Rheumatol Adv Pract. 2024 Jul 26;8(3):rkae087. doi: 10.1093/rap/rkae087 (PMC11333759; doi:10.1093/rap/rkae087)
Supplement: rkae087_Supplementary_Data [file rkae087_supplementary_data.zip › STROBE BAxSIC.docx]

STROBE Statement—checklist of items that should be included in reports of observational studies

|  | Item No. | Recommendation | Page  No. | Relevant text from manuscript |
| --- | --- | --- | --- | --- |
| **Title and abstract** | 1 | (*a*) Indicate the study’s design with a commonly used term in the title or the abstract | 1 | “British Axial Spondyloarthritis Inception Cohort (BAxSIC): A protocol for a multicentre real world observational cohort study of early axial Spondyloarthritis.” |
|  |  | (*b*) Provide in the abstract an informative and balanced summary of what was done and what was found | 2 | See abstract |
| Introduction | | | |  |
| Background/rationale | 2 | Explain the scientific background and rationale for the investigation being reported | 4 | “Diagnostic delay is common in axSpA, and worse in females than males. In the UK, a recent report from the National Axial Spondyloarthritis Society (NASS) highlighted a mean time from symptom onset to diagnosis of 8.5 years. Causes of diagnostic delay in axSpA are multifactorial and include the insidious onset of the disease, poor public and healthcare worker awareness, the absence of diagnostic serum biomarkers and the reliance on clinician diagnosis based on the history, imaging and laboratory findings. The impact of this delay upon patients is poorly researched, with few small-scale studies, primarily focusing on male patients with r-axSpA.. A recent study showed a higher burden of disease with the development of uveitis and inflammatory bowel disease associated with longer diagnostic delay. Further research into the impact of diagnostic delay on the individual’s initial presentation and on the wider axSpA population is therefore vital to improving patient care and outcomes.” |
| Objectives | 3 | State specific objectives, including any prespecified hypotheses | 4 | “Hypothesizing that diagnostic delay leads to worse long-term functional outcomes and affects the overall disease course of axSpA, we aimed to assess the impact of diagnostic delay on clinical presentation and long-term outcomes, including work participation, quality of life and function in axSpA.” |
| Methods | | | |  |
| Study design | 4 | Present key elements of study design early in the paper | 5 | “The BAxSIC study (https://baxsic.uk) is a prospective inception cohort study, recruiting patients with newly diagnosed axSpA, from secondary and tertiary rheumatology centres across the UK.” |
| Setting | 5 | Describe the setting, locations, and relevant dates, including periods of recruitment, exposure, follow-up, and data collection | 5-6 | “Study design  The BAxSIC study (https://baxsic.uk) is a prospective inception cohort study, recruiting patients with newly diagnosed axSpA, from secondary and tertiary rheumatology centres across the UK.”  “Follow up procedures  At 6 monthly intervals (months 6, 12, 18, 30 and 36 participants will be asked to complete electronic data collection sheets and questionnaires (Table 1) via email or text message. These will collect data regarding disease activity and EMMs in addition to patient-reported outcome measures and questionaries. Participants may request paper versions, if required.  At 24 months (Figure 1), participants will be invited for a further in-person review to collect data on medication usage related to axSpA, employment status, alcohol, and smoking status and co-morbidities. Participants will be examined for any extra-axial features of Spondyloarthritis and data from routinely performed MRI, X-ray and CRP/ESR will be collected. Patient reported outcomes and questionnaire data will also be collected at this visit” |
| Participants | 6 | (*a*) *Cohort study*—Give the eligibility criteria, and the sources and methods of selection of participants. Describe methods of follow-up  *Case-control study*—Give the eligibility criteria, and the sources and methods of case ascertainment and control selection. Give the rationale for the choice of cases and controls  *Cross-sectional study*—Give the eligibility criteria, and the sources and methods of selection of participants | 5-6 | “Patients are primarily recruited from rheumatology clinics. Eligible participants are consecutive patients who are >16 years of age with a diagnosis of axSpA made within the last 6 months prior to recruitment by a consultant rheumatologist, and able to give informed consent. The exclusion criteria are age <16, unable to provide informed consent in English (despite efforts to facilitate informed consent through a Trust appointed interpreter) or deemed, in any other way, to be unable to give informed consent. Recruitment to this study is based on a collaborative effort between multiple UK secondary and tertiary centres with the help of BRITSpA. Recruitment will last for 3 years with a total follow up duration of 3 years (36 months)” |
|  |  | (*b*) *Cohort study*—For matched studies, give matching criteria and number of exposed and unexposed  *Case-control study*—For matched studies, give matching criteria and the number of controls per case |  | NA |
| Variables | 7 | Clearly define all outcomes, exposures, predictors, potential confounders, and effect modifiers. Give diagnostic criteria, if applicable | 6 (and table 1) | *“Participants will complete questionnaires to assess function, work participation, disease activity, fatigue, co-morbidities, overall health, and quality of life”*  *“These will collect data regarding disease activity and EMMs in addition to patient-reported outcome measures and questionaries.”*  *“At 24 months (Figure 1), participants will be invited for a further in-person review to collect data on medication usage related to axSpA, employment status, alcohol, and smoking status and co-morbidities. Participants will be examined for any extra-axial features of Spondyloarthritis and data from routinely performed MRI, X-ray and CRP/ESR will be collected. Patient reported outcomes and questionnaire data will also be collected at this visit”* |
| Data sources/ measurement | 8* | For each variable of interest, give sources of data and details of methods of assessment (measurement). Describe comparability of assessment methods if there is more than one group | *5-6* | *“Participants will complete questionnaires to assess function, work participation, disease activity, fatigue, co-morbidities, overall health, and quality of life”*  *“These will collect data regarding disease activity and EMMs in addition to patient-reported outcome measures and questionaries.”*  *“At 24 months (Figure 1), participants will be invited for a further in-person review to collect data on medication usage related to axSpA, employment status, alcohol, and smoking status and co-morbidities. Participants will be examined for any extra-axial features of Spondyloarthritis and data from routinely performed MRI, X-ray and CRP/ESR will be collected. Patient reported outcomes and questionnaire data will also be collected at this visit”* |
| Bias | 9 | Describe any efforts to address potential sources of bias |  | NA |
| Study size | 10 | Explain how the study size was arrived at | 5 | *“There is no minimum sample size required for the study due to the exploratory nature of the primary outcome.”* |

Continued on next page

| Quantitative variables | 11 | Explain how quantitative variables were handled in the analyses. If applicable, describe which groupings were chosen and why |  | NA |
| --- | --- | --- | --- | --- |
| Statistical methods | 12 | (*a*) Describe all statistical methods, including those used to control for confounding |  | NA |
|  |  | (*b*) Describe any methods used to examine subgroups and interactions |  | NA |
|  |  | (*c*) Explain how missing data were addressed |  | NA |
|  |  | (*d*) *Cohort study*—If applicable, explain how loss to follow-up was addressed  *Case-control study*—If applicable, explain how matching of cases and controls was addressed  *Cross-sectional study*—If applicable, describe analytical methods taking account of sampling strategy |  | NA |
|  |  | (*e*) Describe any sensitivity analyses |  | NA |
| Results | | | | |
| Participants | 13* | (a) Report numbers of individuals at each stage of study—eg numbers potentially eligible, examined for eligibility, confirmed eligible, included in the study, completing follow-up, and analysed |  | NA |
|  |  | (b) Give reasons for non-participation at each stage |  | NA |
|  |  | (c) Consider use of a flow diagram |  | NA |
| Descriptive data | 14* | (a) Give characteristics of study participants (eg demographic, clinical, social) and information on exposures and potential confounders |  | NA |
|  |  | (b) Indicate number of participants with missing data for each variable of interest |  | NA |
|  |  | (c) *Cohort study*—Summarise follow-up time (eg, average and total amount) |  | NA |
| Outcome data | 15* | *Cohort study*—Report numbers of outcome events or summary measures over time |  | *NA* |
|  |  | *Case-control study—*Report numbers in each exposure category, or summary measures of exposure |  |  |
|  |  | *Cross-sectional study—*Report numbers of outcome events or summary measures |  |  |
| Main results | 16 | (*a*) Give unadjusted estimates and, if applicable, confounder-adjusted estimates and their precision (eg, 95% confidence interval). Make clear which confounders were adjusted for and why they were included |  | NA |
|  |  | (*b*) Report category boundaries when continuous variables were categorized |  | NA |
|  |  | (*c*) If relevant, consider translating estimates of relative risk into absolute risk for a meaningful time period |  | NA |

Continued on next page

| Other analyses | 17 | Report other analyses done—eg analyses of subgroups and interactions, and sensitivity analyses |  | NA |
| --- | --- | --- | --- | --- |
| Discussion | | | | |
| Key results | 18 | Summarise key results with reference to study objectives |  | NA |
| Limitations | 19 | Discuss limitations of the study, taking into account sources of potential bias or imprecision. Discuss both direction and magnitude of any potential bias | 9 | “However, this minimally disruptive approach may lead some participants to disengage from the study and therefore fail to complete the virtual questionnaires, therefore encouraging ongoing participant is vital to the study’s success. A previous axSpA cohort study utilising a virtual follow up to capture outcomes in axSpA has been successfully performed in the UK with good rates of follow-up data completion (19). Yet, a number of considerations were taken at the time of study design in order to minimise attrition over time. These include the short time to completion of virtual visits which averages 10 minutes, and a window of ±14 days at each end of the follow-up visit's target providing a total of 28 days for visit completion. In addition, up to 3 electronic reminders are sent automatically by the REDCap system including a standard e-mail containing the survey link and unique QR code. The study has recently been granted ethical approval for the use of telephone text message and remote consent which is also expected to minimise disruption and encourage engagement from study participants. A dedicated section for participants within the study website includes an easy-to-understand timeline (https://baxsic.uk/patient-involvement-in-baxsic/).” |
| Interpretation | 20 | Give a cautious overall interpretation of results considering objectives, limitations, multiplicity of analyses, results from similar studies, and other relevant evidence |  | NA |
| Generalisability | 21 | Discuss the generalisability (external validity) of the study results |  | NA |
| Other information | |  | | |
| Funding | 22 | Give the source of funding and the role of the funders for the present study and, if applicable, for the original study on which the present article is based | 14-15 | “Competing interests” |

*Give information separately for cases and controls in case-control studies and, if applicable, for exposed and unexposed groups in cohort and cross-sectional studies.

**Note:** An Explanation and Elaboration article discusses each checklist item and gives methodological background and published examples of transparent reporting. The STROBE checklist is best used in conjunction with this article (freely available on the Web sites of PLoS Medicine at http://www.plosmedicine.org/, Annals of Internal Medicine at http://www.annals.org/, and Epidemiology at http://www.epidem.com/). Information on the STROBE Initiative is available at www.strobe-statement.org.
